# Prevalence, Risk Factors, and Economical Cost of Work-Related Injuries Among Olive Workers in the Achaia Region, Greece

**DOI:** 10.7759/cureus.39657

**Published:** 2023-05-29

**Authors:** Pantelis Politis, Panagiotis Lepetsos, Eleni Jelastopulu, Panagiotis Megas, Michalis Leotsinidis

**Affiliations:** 1 Department of Public Health, School of Medicine, University of Patras, Patras, GRC; 2 Department of Orthopedics, Athens Medical Center, Athens, GRC; 3 Department of Orthopedics, School of Medicine, University of Patras, Patras, GRC

**Keywords:** occupational health, financial cost, risk factors, olive gathering, farm-related injuries

## Abstract

Background

Olive gathering involves tree climbing, carrying heavy loads, navigating rough terrain, and using sharp tools. However, little is known about occupational injuries among olive workers. The aim of this study is to evaluate the prevalence and risk factors of occupational injuries among olive workers in a rural Greek area and to assess the financial burden on the health system and insurance funds.

Methods

A questionnaire was administered to 166 olive workers in the Aigialeia municipality in the Achaia region, Greece. The questionnaire contained detailed information on demographic characteristics, medical history, working environment, protective measures, gathering tools, and type and site of injuries. Moreover, data were recorded about the duration of hospitalization, medical examinations and treatment received, sick leaves, complications, and rate of re-injury. Direct economic costs were calculated for hospitalized and non-hospitalized patients. The associations between olive workers’ characteristics, risk factors, and occupational injury within the last year were examined using log-binomial regression models.

Results

In total, 85 injuries were recorded in 50 workers. The prevalence of one or more injuries in the last year was 30.1%. Factors associated with a higher rate of injury were male gender, age > 50 years, working experience > 24 years, history of arterial hypertension and diabetes mellitus, climbing habits, and non-use of protective gloves. The average cost of agricultural injuries was more than 1400 € per injury. The cost seems to be associated with the severity of the injury, as injuries requiring hospitalization were associated with increased costs, higher cost of medication, as well as more days of sick leave. Losses due to sick leave cause the greatest financial costs.

Conclusions

Farm-related injuries are quite usual among olive workers in Greece. Injury risk is influenced by gender, age, working experience, medical history, climbing habits, and use of protective gloves. Days off work have the greatest financial cost. These findings can be useful as a starting point to train olive workers to reduce the incidence of farm-related injuries in Greece. Knowledge of risk factors for farm-related injuries and diseases could help the development of proper interventions to minimize the problem.

## Introduction

The agricultural sector includes a wide range of occupational hazards with remarkable morbidity and mortality. Engaging in agricultural work is directly related to health and safety due to exposure to the natural environment, close contact with plants, difficult postures, and the use of potentially dangerous agricultural tools and machinery [1]. Farmers are exposed to difficult conditions and perform jobs without the presence of other people, without first aid, and in places inaccessible throughout the day without a specific timetable [2-4].

Greece is a country with a mild climate and the nature of agriculture is similar to that of other Southern European countries. Greece is one of the world's leading producers and exporters of olive oil. Olive oil is an important agricultural product of the country with great medical benefits and also an important part of the Greek economy [5]. From antiquity to the present day, olive oil has played an important role in the economic, rural, and cultural life of the place. Greece is the third highest olive oil producer in the world, after Spain and Italy with annual production ranging from 250,000 to 400,000 tons of olive oil, depending on the olive oil year [6].

The process of olive gathering involves the careful collection of olives from the trees, which are then processed for oil extraction. Olives are harvested either manually or mechanically. In hand harvesting, olives are carefully picked by hand, using small rakes or combs to gently comb the branches and remove the fruit. Hand harvesting is applied to table olives, to avoid damage to fruit and quality degradation. In mechanical harvesting, mechanical harvesters, also known as shakers or vibrators, are used to shake the olive trees, causing the ripe olives to fall onto nets or collection systems that have previously been laid down under the olive trees. These machines can be tractor-mounted or self-propelled and are equipped with rotating rods or paddles that gently shake the branches. While mechanical harvesting is faster and more efficient, it may result in a higher percentage of damaged olives and can be less suitable for certain tree varieties or terrains. After harvesting, the olives are washed to remove dirt, leaves, and twigs. After the twigs are filtered out with grids, the fruit is ready for processing into oil [7].

Literature data indicate that farm workers suffer from a plethora of occupational injuries with a high socio-economic cost [8]. The injury risk for olive workers can vary depending on several factors, including the type of work being performed, the work environment, the use of machinery and tools, the level of training and experience of the workers, and adherence to safety protocols. While olive harvesting is generally considered a low-risk activity compared to some other agricultural tasks, there are still potential hazards that can lead to injuries, including falls and strains, cuts and puncture wounds, machinery-related injuries, and heat-related illnesses. Usual agricultural injuries include lacerations, head injuries, hand injuries, eye injuries, fractures, and falls; however, no data is available on farm injuries of olive workers in Greece [1]. The aim of this study is to evaluate the prevalence and the risk factors of occupational injuries among olive workers during olive harvesting and collection in a rural Greek district and to assess the financial burden on the health system and insurance funds. The results of this study may shed light on the unknown epidemiology of injuries during the olive harvest. The identification of the emerging risk factors may be used to warn farmers and the possible modification of aggravating factors that increase the risk of accidents. The results of the study can be the basis for future preventive medicine programs helping to design a proper and safe agricultural policy.

## Materials and methods

In this cross-sectional study, data were collected from olive workers from the Aigialeia municipality in the Achaia region, a rural area in western Greece, from November 2011 to March 2012 via questionnaire. Inclusion criteria included: (1) employment as an olive worker on a study plantation and (2) more than one year of experience working as an olive worker. This study was approved by the Institutional Review Boards at the School of Medicine, University of Patras (Approval number: 124/2012). Written informed consent was obtained from all participants of the study.

The questionnaire was initially distributed to 40 olive growers who were not included in the final sample of the study, in order to assess whether it is understandable, find possible omissions, and whether the query structure responds to actual working conditions. The questionnaire contained detailed information on demographic characteristics, medical history, previous injuries, injuries < 12 months ago, work environment, protective measures, gathering tools, and type and site of injuries. Demographic characteristics (independent variables) included in the questionnaire were gender (male/female), age (years), height (cm), weight (kg), nationality (Greek/other), work experience (years), work formal relationship (farm owner or employee), training on work safety (yes/no), and social insurance (yes/no). Medical information included the history of arterial hypertension, diabetes mellitus, and joint diseases. Data about working habits included working hours, breaks, lunches, alcohol consumption, tree climbing, use of protective gloves/glasses/boots, use of gathering tools (ladders, olive harvesters, poles, sticks, olive harvesting machines, pruning scissors, cutters, saw, and chainsaw).

Farm injury was defined as any injury that happens on the farm (outside the home) to farm workers, non-working farm residents, and visitors. When a person had suffered multiple injuries, the more serious one was considered as the index injury. Information about work-related injuries in the previous 12 months was also collected, along with the site and type of injuries and medical treatment. Moreover, data were recorded about the duration of hospitalization, medical examinations, and the medication they received in the hospital, along with complications and rate of re-injury. The days of absence from work were also recorded based on the instructions received from treating physicians. For the estimation of the in-hospital cost, we took into account the Greek version of the diagnosis-related group (DRG) reimbursement system (KEN), according to the Greek law Β’940/27-3-2012. Table 1 shows the considered costs of medical visits, pharmaceutical treatment, medical tests, physical therapy, and sick leaves. 

**Table 1:** Considered mean costs in Euros (€)

Event/Activity	Considered mean cost
Physician examination	50 € / visit
Complete blood count (CBC)	2 € / CBC
X-rays	7 € / x-ray
Computed tomography (CT)	70 € / CT
Ultrasound (US)	40 € / US
Magnetic resonance imaging (MRI)	230 € / MRI
Pharmaceutical treatment	10 € / day
Physical therapy	15 € / session
Sick leave	60 € / day

For the hospitalized patients, the total cost was calculated by the sum of the KEN cost, the cost of pharmaceutical treatment, the cost of physical therapy, and the cost of sick leave. For the non-hospitalized patients, the total cost was calculated by the sum of the cost of medical visits, the cost of medical tests, the cost of pharmaceutical treatment, the cost of physical therapy, and the cost of sick leave.

Statistical analysis

The data were collected on paper and de-identified data were entered into Microsoft Excel (Microsoft Corporation, Redmond, Washington, United States) for storage. Statistical analysis was conducted using the statistical package PASW Statistics 18 (SPSS Inc., Chicago, Illinois, United States). The descriptive characteristics of the sample were originally calculated. For the quantitative variables, the mean and the standard deviation (SD) were calculated. For the categorical variables, the frequencies expressed in absolute values ​​and percentages were calculated. All demographic variables (independent variables) were compared to the dependent variable (farm-related injury during the year under study) using χ^2^ test. The logistic regression model was used to estimate the odds ratio (OR) of the injury event and to identify independent risk factors associated with the injury. An acceptable level of statistical significance was set at 5% (p <0.05), while confidence intervals were calculated at 95%.

## Results

Descriptive characteristics of the studied population are shown in Table 2. A total of 166 olive workers completed the questionnaire. Participants ranged between 19 and 86 years old, with a mean age of 49.7. Olive workers had worked an average of 26.1 years in the industry, with a range of 1-70 years.

**Table 2:** Descriptive characteristics of the studied population

Characteristics	Value
Cases (n)	166
Male (%)	81.3%
Mean age (years)	49.7 (range 19 – 86)
Mean height (cm)	171.8 (range 150 – 194)
Mean weight (kg)	82.2 (range 50 – 130)
Nationality	98.2% Greek
Land property	75.3% Land owner
	24.7% Worker
Mean work experience (years)	26.1 (range 1 – 70)
Mean duration of daily work (hours)	7.77 (range 1 – 14)
Social insurance	90.4% Secured
	9.6% Insecured

Regarding the medical history, 13.3% of the participants (n = 22) suffered from arterial hypertension, 4.8% (n = 8) suffered from diabetes mellitus, and 10.2% (n = 17) suffered from joint diseases. Most workers had never received training on work safety (71.7%). Only 26.5% (n = 44) worked more than eight hours, while 86.8% (n = 144) had a break during work with a mean duration of 21.5 minutes. Of the participants, 75.9% (n = 126) ate food during work while 13.3% (n = 22) consumed alcohol. Table 3 shows the working habits and protective tools of the participants.

**Table 3:** Working habits of the studied population

Working habit	Rate (%)
Use of protective gloves	66.7%
Use of protective glasses	29.9%
Use of protective boots	47.0%
Use of ladder	72.1%
Tree climbing	75.0%
Use of scallops	47,6%
Use of poles	31,9%
Use of sticks	40.9%
Use of olive harvesting machines	59.6%
Use of pruning scissors	30.1%
Use of cutters	13.9%
Use of saw	56.0%
Use of chainsaw	75.9%

About one-third (30.1%, n = 50) of the participants reported a new injury in the last 12 months. Total number of injuries was 85. Most commonly, the injury happened during olive harvesting (44.1%). Of the injuries, 56.5% were blunt injuries, 27.1% were fractures, and 16.4% were sprains. The most common affected site was the abdominal area (41.2%, n = 35), followed by the thoracic wall (29.4%, n = 25) and head (8.2%, n = 7). The rate of tendon rupture was 39%. Seven percent (n = 6) of the injured workers needed to be transferred by ambulance and 20.0% (n = 17) was hospitalized. For treatment of injuries, 54.1% of the participants were sutured, 30.6% were treated with a cast, 11.8% were treated with a splint, and 3.5% needed an operation; 65.88% of the injuries were treated in the public hospital, 17.65% by a private doctor, and 16.47% in a rural health center. Of the injured workers, 58.8% (n = 50) had to receive medication after their injury and 12.9% (n = 11) needed physical therapy. The mean duration of absence from work due to injury was 16.68 days (range 0-360 days). The median duration of absence from work was five days. Of the injured, 94.1% did not show any complications after the initial injury while 35.29% suffered some kind of re-injury after the initial injury. Table 4 summarized the mean and total costs for hospitalized and non-hospitalized patients. 

**Table 4:** Cost analysis according to patient hospitalization in Euro (€)

	Hospitalized	Non-hospitalized	Total
Total cost of visits to private physicians	0	750	750
Mean cost of visits to private physicians	0	11,02	11,02
Total cost of ΚΕΝ	19488	0	19488
Mean cost of ΚΕΝ	1146	0	1146
Total cost of medical examinations	0	497	497
Mean cost of medical examinations	0	7,3	7,3
Total cost of medical operations	0	9800	9800
Mean cost of medical operations	0	144,1	144,1
Total cost of pharmaceutical treatment	1650	2400	4050
Mean cost of pharmaceutical treatment	97,05	35,29	47,64
Total cost of physical therapy	1335	450	1785
Mean cost of physical therapy	78,52	6,61	21
Total cost of sick leaves	37620	47460	85080
Mean cost of sick leaves	2213	698	1001
TOTAL COST	60093	61357	121450
MEAN COST	3535	902	1429

For the hospitalized patients, the average duration of hospitalization was 4.53 ± 2.5 days (range 0-15 days). Among the hospitalized patients, 15 needed medication after being discharged from the hospital. The mean duration of medication was 9.7 days. A total of seven patients needed physical therapy after being discharged from the hospital. A total of 89 physiotherapy sessions were needed. Also, the treated patients received a total of 627 days of sick leave. As shown in Table 4, the 17 patients who were hospitalized had a total injury cost of 60,093 € while the average cost per injured patient was 3535 €.

A total of 68 injured workers received outpatient treatment. Among the 15 patients who were treated by private physicians, the total number of medical visits was 15 and the total financial cost was 750 € (range 50-100 €/patient). Among the 68 non-hospitalized patients, five patients underwent blood tests, 21 patients underwent x-rays, one patient underwent computed tomography (CT), one patient underwent ultrasound, and one patient underwent magnetic resonance imaging (MRI). A cast or splint was placed in four patients. The mean duration of pharmaceutical treatment was 3.5 days. A total of 30 physiotherapy sessions were needed. Also, the non-hospitalized patients received a total of 791 days of sick leave. As shown in Table 4, the 68 non-hospitalized patients had a total cost of injury of 61,357 € while the average cost per injury was 902 €.

A total of 51 patients received in-home medication, with the total cost reaching 4050 € and the average cost of pharmaceutical treatment reaching 47.64 € (range 20-300 €). A total of 12 patients needed physiotherapy, with a total of 119 hours. The total cost of physiotherapy reached 1785 € and the average cost of physiotherapy per patient was 21 €. A total of 56 patients received sick leave. The average duration of sick leave was 16.7 days. The total cost of sick leave reached 85,080 €, with the average cost of sick leave per patient reaching 1001 €. Finally, the total cost of agricultural injuries in the present study reached 121,450 €. The average cost of agricultural injuries in the present study is 1429 € (Table 4).

As shown in Table 5, univariate analysis of the risk factors among olive workers showed that male gender (p = 0.021), age > 50 years (p = 0.009), arterial hypertension (p = 0.029), diabetes mellitus (p = 0.005), working experience > 24 years (p = 0.007), and tree climbing (p = 0.011) are associated with a higher risk of farm-related injuries among olive workers. The use of protective gloves (p = 0.009) has been correlated with a decreased incidence of occupational injury. No association was observed in relation to weight, height, nationality, land property, social insurance, working habits, use of protective glasses and boots, and olive gathering techniques (p > 0.05).

**Table 5:** Association of occupational injuries in the Aigialeia municipality in the Achaia region of Greece with independent variables

Variable		Occupational injury n (%)		P-value
		No	Yes	
Gender	Male	89 (53.6%)	46 (27.7%)	0.021
	Female	27 (16.3%)	4 (2.4%)	
Nationality	Greek	114 (68.7%)	49 (29.5%)	> 0.05
	Non-Greek	2 (1.2%)	1 (0.6%)	
Land property	Worker	29 (17.5%)	12 (7.2%)	> 0.05
	Land owner	87 (52.4%)	38 (22.9%)	
Age	< 50 years	65 (39.2%)	17 (10.2%)	0.009
	> 50 years	51 (30.7%)	33 (19.9%)	
Height	< 170 cm	66 (39.8%)	24 (14.5%)	> 0.05
	> 170 cm	50 (30.1%)	26 (15.7%)	
Weight	< 80 kg	61 (36.8%)	24 (14.5%)	> 0.05
	> 80 cm	55 (33.1%)	26 (15.7%)	
Social insurance	Yes	102 (61.5%)	48 (28.9%)	> 0.05
	No	14 (8.4%)	2 (1.2%)	
Previous injury (3 years ago)	Yes	35 (21.1%)	18 (10.8%)	> 0.05
	No	81 (48.8%)	32 (19.3%)	
Arterial Hypertension	Yes	11 (6.6%)	11 (6.6%)	0.029
	No	105 (63.3%)	39 (23.5)	
Diabetes mellitus	Yes	2 (1.2%)	6 (3.6%)	0.005
	No	114 (68.7%)	44 (26.5%)	
Joint disease	Yes	12 (7.2%)	5 (3.0%)	> 0.05
	No	104 (62.6%)	45 (27.1%)	
Training courses	Yes	33 (19.9%)	14 (8.4%)	> 0.05
	No	83 (50.0%)	36 (21.7%)	
Working experience	< 24 years	66 (39.7%)	17 (10.2%)	0.007
	> 24 years	50 (30.1%)	33 (19.9%)	
Work Break	Yes	100 (60.2%)	16 (9.6%)	> 0.05
	No	44 (26.5%)	6 (3.6%)	
Lunch at Work	Yes	91 (54.8%)	35 (21.1%)	> 0.05
	No	25 (15.1%)	15 (9.0%)	
Alcohol at Work	Yes	12 (7.3%)	10 (6.1%)	> 0.05
	No	103 (62.4%)	40 (24.2%)	
Use of protective gloves	Yes	85 (51.5%)	25 (15.2%)	0.009
	No	30 (18.2%)	25 (15.2%)	
Use of protective glasses	Yes	34 (20.7%)	15 (9.2%)	> 0.05
	No	80 (48.8%)	35 (21.3%)	
Use of protective boots	Yes	53 (32.3%)	24 (14.6%)	> 0.05
	No	61 (37.2%)	26 (15.9%)	
Use of ladder	Yes	82 (49.7%)	37 (22.4%)	> 0.05
	No	33 (20.0%)	13 (7.9%)	
Climbing on tree	Yes	79 (48.2%)	44 (26.8%)	0.011
	No	35 (21.3%)	6 (3.7%)	
Use of olive harvesters	Yes	57 (34.3%)	22 (13.3%)	> 0.05
	No	59 (35.5%)	28 (16.9%)	
Use of poles	Yes	35 (21.1%)	18 (10.8%)	> 0.05
	No	81 (48.8%)	32 (19.3%)	
Use of sticks	Yes	49 (29.5%)	19 (11.5%)	> 0.05
	No	67 (40.4%)	31 (18.7%)	
Use of olive harvesting machines	Yes	66 (39.8%)	33 (19.9%)	> 0.05
	No	50 (30.1%)	17 (10.2%)	
Use of pruning scissors	Yes	35 (21.1%)	15 (9.0%)	> 0.05
	No	81 (48.8%)	35 (21.1%)	
Use of cutters	Yes	15 (9.0%)	8 (4.8%)	> 0.05
	No	101 (60.8%)	42 (25.3%)	
Use of saw	Yes	64 (38.6%)	29 (17.5%)	> 0.05
	No	52 (31.3%)	21 (12.6%)	
Use of chainsaw	Yes	88 (53.0%)	38 (22.9%)	> 0.05
	No	28 (16.9%)	12 (7.2%)	

Table 6 shows the results of the multivariate regression analysis of the studied risk factors. Male gender (p = 0.027), age > 50 years (p = 0.01), diabetes mellitus (p = 0.046), working experience > 24 years (p = 0.008), no use of protective gloves (p = 0.003), and tree climbing (p = 0.014) are associated with a higher risk of agricultural injuries among olive workers.

**Table 6:** Odds ratio (OR) and 95% confidence intervals (CI) with p determined by logistic regression for the explanatory variables in relation to the outcome variable injury

Variable	OR	95% CI	p-value
Male gender	1.28	1.092 – 1.866	0.027
Age > 50 years	2.51	1.242 – 5.056	0.01
Diabetes mellitus	5.67	1.030 – 31.19	0.046
Working experience > 24 years	2.56	1.284 – 5.113	0.008
No use of protective gloves	2.83	1.417 – 5.667	0.003
Tree climbing	1.31	1.120 – 1.789	0.014

## Discussion

Occupational farm injuries represent an under-appreciated public health problem the magnitude of which increases with the prevalence of farming in a particular population. Farm workers are continually exposed to physical, chemical, and biological hazards [1]. The current study provides a baseline for injury prevalence among olive workers in rural areas in Greece, and an investigation of the potential risk factors associated with occupational injuries. To the best of our knowledge, this is the first study investigating the prevalence and the associated risk factors for farm-related injuries during olive gathering in a rural Greek district.

According to official statistics, agricultural accidents in Greece are twice as high as occupational accidents. Every year, more than 38,000 agricultural accidents are recorded in Greece, while the corresponding work accidents reach 16,800 [9]. The main causes of injury to farmers are falls and accidents with tools or agricultural machinery. In the agricultural industry, falls are generally reported as the second leading cause of death after motor vehicle accidents. The vast majority (90%) of the injured workers are Greeks, with 70% of injuries involving men [1].

Olive gathering requires olive workers to climb olive trees, and work with cutting and piercing instruments, putting them at risk for occupational injuries. This study found the 12-month prevalence of farm-related injuries in the olive gathering industry to be 30.1%. Similar studies in Greek populations are very scarce. Frantzeskou et al. reported a 28% incidence of occupational injuries among Greek fishermen [10]. One of the conclusions of this study is that men are more predisposed to occupational injuries. Several studies have found similar correlations between gender and occupational injuries [11-13]. This association can be attributed to the fact that men dedicate more hours to the farm than women do, exposing themselves to several hazards (machinery, tractors, hand tools, etc.) [14]. Furthermore, age and working experience have been associated with farm-related injuries. The mean age of our study population is about 50 years old and the mean working experience is 26 years. As age increases, cognitive functions may be impaired making elderly workers more vulnerable to injuries. Nevertheless, many Greek pensioners are occasionally occupied in olive gathering, increasing the mean age of the studied population.

Assessment of the financial cost of agricultural injuries during olive harvesting was a goal of this study. The cost seems to be associated with the severity of the injury, as injuries requiring hospitalization were associated with increased costs, higher cost of medication, as well as more days of sick leave. These injuries can result in medical expenses, lost productivity, decreased labor availability, and increased insurance costs. The financial burden extends to injured individuals, their families, healthcare systems, and even the wider economy. Losses due to sick leave cause the greatest financial costs. The correlation of financial costs with the severity of agricultural injury has recently emerged in the literature [15].

This study concluded that the use of protective gloves decreases significantly the incidence of occupational injuries. However, no correlation was found in relation to the use of protective glasses and boots. Literature suggests that protective measures can improve safety in occupational settings [16]. Hypertension and diabetes were associated with injuries during olive harvest. This can be attributed to the fact that diabetic and hypertensive patients are more likely to have episodes of dizziness and hypoglycemia, which increase the incidence of falls.

In our study, the abdominal area was the most common injured site. Literature has shown that hands are the most affected body part, in farm-related injuries, as they are involved in using machinery and hand tools [11,17]. Moreover, we have concluded that tree climbing is a predisposing factor for occupational injury. As olive gathering often needs tree climbing, falling from a height may be a common way of injury and in this case, the abdomen may be injured. In contrast to other studies, no traffic injuries were reported, since olive gathering does not require the use of specific vehicles.

Agricultural injuries, whether occupational or non-occupational, are a very important issue in the countries of the European Union and must be tackled effectively. Agricultural injuries are severe in terms of morbidity and mortality and there is an urgent need for the European Commission to make significant efforts to prevent them. An early warning system for monitoring work-related injuries could be set up through the network of national agricultural data systems. European training programs focusing on farm safety should also be considered. These could be two ambitious monitoring measures that have the potential to further address this important issue of the European Union and significantly reduce rural injury. At the European level, recommendations should be made for the implementation of specific protocols and forms in the medical environment for the identification and recording of farm injuries. Unified high-quality coding systems at the European Union level should be used, which is a prerequisite for the study and comparison of injury data from different countries.

This study has some limitations. The present study is cross-sectional, including recall bias, making it difficult to justify the causation for many associations. A longitudinal study would be useful. Some indirect costs for medical care, including travel, stay, and food were not assessed. Random sampling was not applied, as contact lists were not available for workers. Furthermore, the study sample was taken from a specific area of a rural district in a Greek province, and the generalization of conclusions must be made with caution.

## Conclusions

Agricultural section is of high importance in the Greek economy and the olive industry is a large part of the agricultural industry. This study demonstrates the high incidence of occupational injury encountered by olive workers in a rural area of Achaia region, Greece. Injury risk is influenced by gender, age, working experience, history of arterial hypertension and diabetes mellitus, climbing habits, and use of protective gloves. Agricultural injuries during olive harvesting can have significant financial implications for both individuals and the agricultural sector as a whole. Knowledge of risk factors for farm-related injuries and diseases could help the development of proper interventions to minimize the problem. More specifically, recommendations should be established about the use of protective equipment, management of comorbidities, and climbing habits. Special attention should be paid to elderly males with high working experience.
